# Blood Lymphocyte-to-Monocyte Ratio Identifies High-Risk Patients in Diffuse Large B-Cell Lymphoma Treated with R-CHOP

**DOI:** 10.1371/journal.pone.0041658

**Published:** 2012-07-23

**Authors:** Zhi-Ming Li, Jia-Jia Huang, Yi Xia, Jian Sun, Ying Huang, Yu Wang, Ying-Jie Zhu, Ya-Jun Li, Wei Zhao, Wen-Xiao Wei, Tong-Yu Lin, Hui-Qiang Huang, Wen-Qi Jiang

**Affiliations:** 1 State Key Laboratory of Oncology in South China, Guangzhou, China; 2 Department of Medical Oncology, Sun Yat-sen University Cancer Center, Guangzhou, China; 3 Department of Clinical Trial Center, Sun Yat-sen University Cancer Center, Guangzhou, China; 4 Department of Radiation Oncology, Sun Yat-sen University Cancer Center, Guangzhou, China; University of Nebraska - Lincoln, United States of America

## Abstract

**Background:**

Recent research has shown a correlation between immune microenvironment and lymphoma biology. This study aims to investigate the prognostic significance of the immunologically relevant lymphocyte-to-monocyte ratio (LMR), in diffuse large B-cell lymphoma (DLBCL) in the rituximab era.

**Methodology/Principal Findings:**

We analyzed retrospective data from 438 newly diagnosed DLBCL patients treated with rituximab plus cyclophosphamide, doxorubicin, vincristine, and prednisone (R-CHOP) therapy. We randomly selected 200 patients (training set) to generate a cutoff value for LMR by receiver operating characteristic (ROC) curve analysis. LMR was then analyzed in a testing set (n = 238) and in all patients (n = 438) for validation. The LMR cutoff value for survival analysis determined by ROC curve in the training set was 2.6. Patients with low LMR tended to have more adverse clinical characteristics. Low LMR at diagnosis was associated with worse survival in DLBCL, and could also identify high-risk patients in the low-risk IPI category. Multivariate analysis identified LMR as an independent prognostic factor of survival in the testing set and in all patients.

**Conclusions/Significance:**

Baseline LMR, a surrogate biomarker of the immune microenvironment, is an effective prognostic factor in DLBCL patients treated with R-CHOP therapy. Future prospective studies are required to confirm our findings.

## Introduction

Diffuse large B-cell lymphoma (DLBCL), the most common subtype of lymphoid neoplasm, is characterized as an aggressive lymphoma with heterogeneous clinical behaviors [1], [2]. DLBCL accounts for 25–30% of non-Hodgkin lymphoma (NHL) among adults in the west, and it is even more prevalent in developing countries [1], [2], [3]. Immunodeficiency is the most extensively described and one of the strongest risk factors of non-Hodgkin lymphoma [4]. People with congenital or acquired systemic immune suppression are at much higher risk of developing lymphoma [5], [6]. Gene-expression profiling (GEP) studies showed a relationship between lymphoma biology and the host immune system, and suggested that gene signatures related to non-malignant tumor microenvironment played an important role in the clinical outcomes of patients with NHL [7], [8], [9]. The gene expression-based prognostic model in DLBCL patients showed that DLBCL survival outcomes were determined not only by clinical parameters, but also by the genes regulating tumor microenvironment interactions [8].

**Table 1:** Baseline clinical characteristics of patients with diffuse large B-cell lymphoma according to lymphocyte-to-monocyte ratio.

Characteristics	All cases	Training set (n = 200)			Testing set (n = 238)		
		LMR>2.6	LMR≤2.6	*P*	LMR>2.6	LMR≤2.6	*P*
**Age (years)**							
>60	151	43	28	0.388	52	28	0.723
≤60	287	86	43		99	59	
**Gender**							
Male	259	78	42	0.856	87	52	0.745
Female	179	51	29		64	35	
**Ann Arbor Stage**							
I–II	239	81	30	0.005	97	31	<0.001
III–IV	199	48	41		54	56	
**ECOG PS**							
0–1	393	113	68	0.059	144	68	<0.001
≥2	45	16	3		7	19	
**Serum LDH level**							
>245 U/L	170	37	42	<0.001	41	50	<0.001
≤245 U/L	268	92	29		110	37	
**Primary Involved Sites**							
Nodal	190	69	24	0.008	62	35	0.900
Extranodal	248	60	47		89	52	
**Extranodal sites**							
0–1	383	117	60	0.189	134	72	0.193
≥2	55	12	11		17	15	
**IPI score**							
0–1	250	81	29	0.003	104	36	<0.001
2–5	188	48	42		47	51	
**Absolute monocyte count**							
≥0.62×10^9^/L	198	45	47	<0.001	50	56	<0.001
<0.62×10^9^/L	240	84	24		101	31	
**Absolute lymphocyte count**							
>1.10×10^9^/L	354	118	43	<0.001	142	51	<0.001
≤1.10×10^9^/L	84	11	28		9	36	

Abbreviations: LMR, lymphocyte-to-monocyte ratio; ECOG PS, Eastern Cooperative Oncology Group performance status; LDH, lactate dehydrogenase; IPI, international prognostic index.

Although it is tempting to hypothesize that specific gene signatures of host immunity can predict prognosis of DLBCL patients, considerations such as cost and technical limitations make their application on a routine basis impractical. A large number of studies have therefore focused on the search for surrogate biomarkers which are immunologically relevant and can serve as prognostic factors [10], [11]. Lymphopenia, a surrogate marker of immune suppression, was found to predict survival in DLBCL [12], [13]. Monocyte, which are considered immunologically relevant and are regarded as a surrogate marker of the tumor microenvironment, were also recently reported to be a prognostic factor in DLBCL [14]. Although the introduction of rituximab combined with chemotherapy has greatly improved survival outcomes in DLBCL patients [2], [15], [16], there is limited data available on whether monocyte counts have the same prognostic value in DLBCL patients in the rituximab era. This study aimed to investigate the impact of peripheral blood lymphocyte-to-monocyte ratio (LMR) on survival in DLBCL patients receiving rituximab plus cyclophosphamide, doxorubicin, vincristine, and prednisone (R-CHOP) therapy.

**Figure 1:**
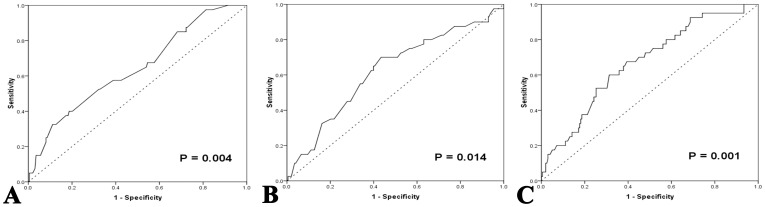
ROC curves analysis for AMC, ALC, and LMR at diagnosis in the training set. A: ROC curves analysis for AMC at diagnosis in the training set. **B:** ROC curves analysis for ALC at diagnosis in the training set. **C:** ROC curves analysis for LMR at diagnosis in the training set. ROC, receiver operating characteristic; AMC, absolute monocyte count; ALC, absolute lymphocyte count; LMR, lymphocyte-to-monocyte ratio.

## Results

### Patient Characteristics

We retrospectively analyzed data from a total of 438 DLBCL patients in this study. The clinical features of all 438 patients, including the training set (n = 200) and the testing set (n = 238), are summarized in Table 1. The median age of all patients at diagnosis was 53 years, (range of 16–86 years). About one half of the patients (239 cases, 54.6%) had localized disease (Ann Arbor stage I–II). Based on the IPI score, 250 patients were in the low-risk group (57.1%), while the remaining 188 cases (42.9%) were in the intermediate or high-risk groups. More than one half of the patients (248 cases, 56.6%) had primary extranodal lymphomas, and 43.4% of the patients (190 cases) had primary nodal lymphomas.

**Figure 2:**
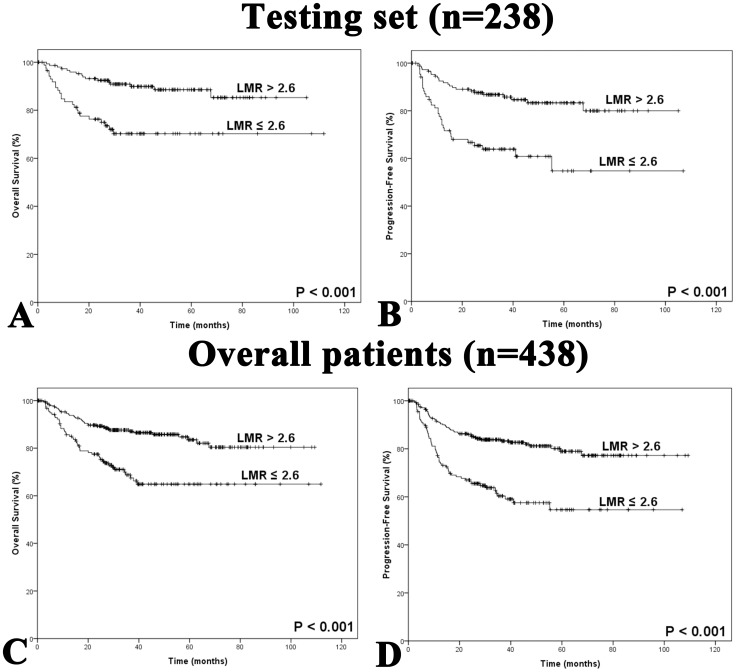
Kaplan-Meier survival analysis of LMR at diagnosis in patients with diffuse large B-cell lymphoma. A: Overall survival according to baseline LMR in the testing set. **B:** Progression-free survival according to baseline LMR in the testing set. **C:** Overall survival according to baseline LMR in all patients. **D:** Progression-free survival according to baseline LMR in all patients. LMR, lymphocyte-to-monocyte ratio.

The absolute monocyte count (AMC) and absolute lymphocyte count (ALC) were derived from pre-treatment CBC counts. The median AMC of all patients at diagnosis, and the 25% and 75% quartiles were 0.60×10^9^/L, 0.40×10^9^/L, and 0.78×10^9^/L, respectively. The median ALC of all patients at diagnosis, and the 25% and 75% quartiles were 1.60×10^9^/L, 1.20×10^9^/L, and 2.20×10^9^/L, respectively. The cutoff points of AMC, ALC, and LMR for survival outcomes were selected by the ROC curve analysis in the training set. The most discriminative cutoff value of AMC was 0.620×10^9^/L, with an area under the curve (AUC) value of 0.642 [95% confidence interval (CI), 0.549–0.736, *P* = 0.004] (Fig. 1A). The most discriminative cutoff value of ALC was 1.095×10^9^/L, with an AUC value of 0.623 (95% CI, 0.526–0.720, *P* = 0.014) (Fig. 1B). ROC curve analysis in the training set established 2.586 as the cutoff point of LMR for survival with an AUC of 0.669 (95% CI, 0.580–0.758, P = 0.001) (Fig. 1C). Based on these results, we selected LMR ≤2.6, AMC ≥0.62×10^9^/L, and ALC ≤1.10×10^9^/L, as the optimal cut-off points for survival analysis in the testing set.

**Figure 3:**
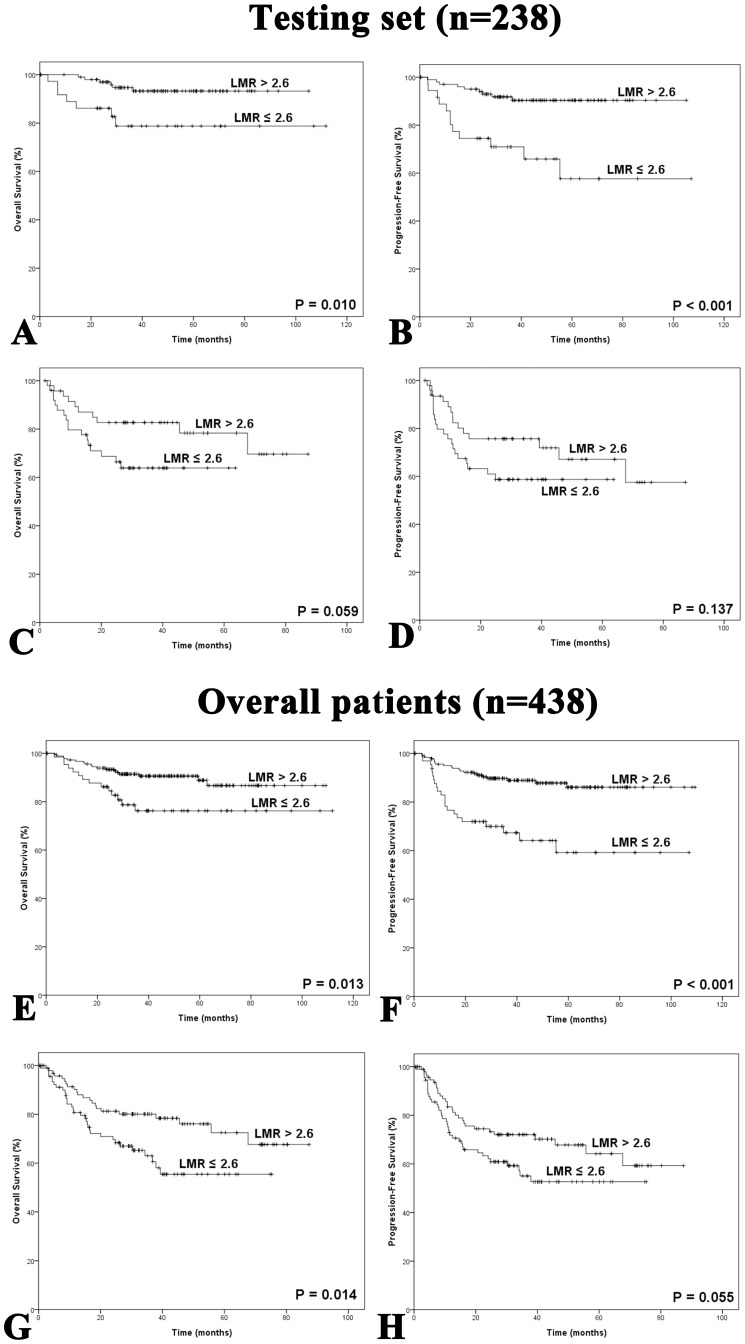
Kaplan-Meier survival analysis of baseline LMR in patients with IPI  = 0–1 or IPI ≥2. **A:** Overall survival of patients with IPI  = 0–1 in the testing set. **B:** Progression-free survival of patients with IPI  = 0–1 in the testing set. **C:** Overall survival of patients with IPI ≥2 in the testing set. **D:** Progression-free survival of patients with IPI ≥2 in the testing set. **E:** Overall survival of patients with IPI  = 0–1 in all patients. **F:** Progression-free survival of patients with IPI  = 0–1 in all patients. **G:** Overall survival of patients with IPI ≥2 in all patients. **H:** Progression-free survival of patients with IPI score ≥2 in all patients. LMR, lymphocyte-to-monocyte ratio; IPI, International Prognostic Index.

The relationships between LMR at the time of diagnosis and baseline clinical features are listed in Table 1. Patients with LMR ≤2.6 had 1) a higher incidence of advanced Ann Arbor stage (*P* = 0.005 for the training set, and P<0.001 for the testing set), 2) elevated LDH level (*P*<0.001 for both sets), and 3) IPI score ≥2 (*P* = 0.003 for the training set, and *P*<0.001 for the testing set). Although low LMR (≤2.6) was significantly related to worse performance status (≥2) in the testing set (*P*<0.001), the training set showed borderline statistical significance (*P* = 0.059). Patients in the training set with primary extranodal lymphomas were significantly more likely to have low LMR at diagnosis (*P* = 0.008), but no statistical significance was observed in the testing set (*P* = 0.900).

**Table 2:** Multivariate analysis of prognostic factors for survival in testing set.

Parameters	OS		PFS	
	RR (95% CI)	*P*	RR (95% CI)	*P*
**Age >60 years**	2.153 (1.038–4.466)	0.039	1.730 (0.933–3.206)	0.082
**Ann Arbor Stage (III–IV)**	1.423 (0.609–3.328)	0.415	2.093 (1.022–4.285)	0.043
**ECOG PS ≥2**	0.992 (0.439–2.240)	0.984	1.005 (0.491–2.057)	0.990
**Serum LDH level >245 U/L**	3.334 (1.403–7.923)	0.006	1.717 (0.842–3.501)	0.137
**Extranodal sites ≥2**	0.898 (0.368–2.194)	0.814	0.828 (0.372–1.844)	0.644
**AMC ≥0.62×10^9^/L**	0.732 (0.325–1.651)	0.452	0.963 (0.487–1.905)	0.913
**ALC ≤1.10×10^9^/L**	0.727 (0.306–1.725)	0.470	0.740 (0.350–1.568)	0.423
**LMR ≤2.6**	3.108 (1.236–7.814)	0.016	2.758 (1.300–5.849)	0.008

Abbreviations: OS, overall survival; PFS, progression-free survival; ECOG PS, Eastern Cooperative Oncology Group performance status; LDH, lactate dehydrogenase; AMC, absolute monocyte count; ALC, absolute lymphocyte count; LMR, lymphocyte-to-monocyte ratio.

### Lymphocyte-to-monocyte Ratio at Diagnosis and Clinical Outcomes

**Table 3:** Multivariate analysis of prognostic factors for survival in all patients with diffuse large B-cell lymphoma.

Parameters	OS		PFS	
	RR (95% CI)	*P*	RR (95% CI)	*P*
**Age >60 years**	1.708 (1.101–2.649)	0.017	1.361 (0.921–2.011)	0.122
**Ann Arbor Stage (III–IV)**	1.032 (0.625–1.705)	0.901	1.785 (1.250–2.769)	0.010
**ECOG PS ≥2**	1.449 (0.872–2.407)	0.152	1.399 (0.888–2.205)	0.148
**Serum LDH level >245 U/L**	2.178 (1.305–3.637)	0.003	1.432 (0.919–2.232)	0.112
**Extranodal sites ≥2**	1.261 (0.695–2.288)	0.445	0.893 (0.514–1.552)	0.688
**AMC ≥0.62×10^9^/L**	1.441 (0.891–2.330)	0.137	1.318 (0.867–2.005)	0.197
**ALC ≤1.10×10^9^/L**	0.984 (0.580–1.670)	0.953	0.952 (0.592–1.533)	0.840
**LMR ≤2.6**	1.669 (1.031–2.702)	0.037	1.877 (1.227–2.872)	0.004

Abbreviations: OS, overall survival; PFS, progression-free survival; ECOG PS, Eastern Cooperative Oncology Group performance status; LDH, lactate dehydrogenase; AMC, absolute monocyte count; ALC, absolute lymphocyte count; LMR, lymphocyte-to-monocyte ratio.

Three hundred and ninety-four (90.0%) of the 438 patients were evaluated for their response to R-CHOP therapy. Treatment response data were available for 211 patients in the testing set (88.7%). Complete remission (CR) was achieved in 153 patients of the testing set, and in 276 patients of the entire series. The CR rate of R-CHOP treatment was significantly higher in patients with LMR >2.6 prior to chemotherapy compared to patients with LMR ≤2.6 at diagnosis (testing set: 78.9% *versus* 61.5%, *P* = 0.006; overall patients: 73.9% *versus* 63.1%, *P* = 0.025). The non-responding (stable disease or progressive disease) rate of R-CHOP treatment seemed to be higher in patients with LMR ≤2.6 than those with LMR >2.6 in all patients (9.2% *versus* 4.0%, *P* = 0.033), but not in the testing set (6.4% *versus* 2.3%, *P* = 0.149).

Kaplan-Meier analysis showed that lower LMR at diagnosis seemed to be associated with inferior overall survival (OS) and progression-free survival (PFS) in the testing set (OS: *P*<0.001; PFS: *P*<0.001; Fig. 2A and 2B). Similar results were observed in the overall set of patients (OS: *P*<0.001; PFS: *P*<0.001; Fig. 2C and 2D). Patients with AMC ≥0.62×10^9^/L had adverse survival outcomes in the testing set (OS: *P* = 0.001; PFS: *P* = 0.009) as well as in the overall set of patients (OS: *P*<0.001; PFS: *P*<0.001). Patients with ALC >1.10×10^9^/L seemed to have significantly better OS and PFS compared to patients with ALC ≤1.10×10^9^/L (testing set: *P* = 0.011 in OS, and *P* = 0.045 in PFS; overall patients: *P* = 0.003 in both OS and PFS).

We showed that in the testing set, patients with a low-risk category of IPI score (IPI  = 0–1), LMR was a useful way to distinguish those with favorable outcomes from those with adverse outcomes (OS: *P* = 0.010; PFS: *P*<0.001; Fig. 3A and 3B). We also showed a similar relationship between LMR and survival in the set of overall patients (OS: *P* = 0.013; PFS: *P*<0.001; Fig. 3E and 3F). In patients with IPI score ≥2, LMR at diagnosis was also helpful in differentiating between patients with different OS, with statistical significance in overall patients (*P* = 0.014, Fig. 3G), and with borderline significance in the testing set (*P* = 0.059, Fig. 3C). The association between LMR and PFS in patients with IPI score ≥2 was marginally significant in overall patients (*P* = 0.055, Fig. 3H), but not in the testing set (*P* = 0.137, Fig. 3D).

### Multivariate Cox Regression Analysis

We used Cox Regression analysis to evaluate the prognostic impact of LMR at diagnosis on the survival of DLBCL patients. Parameters included in the multivariate survival analysis are shown in Table 2 and Table 3. In the testing set, baseline LMR was identified as an independent prognostic factor for OS (relative risk, 3.108; 95% CI, 1.236–7.814; *P* = 0.016; Table 2) and PFS (relative risk, 2.758; 95% CI, 1.300–5.849; *P* = 0.008; Table 2). When adjusted for variables of IPI score, LMR at diagnosis retained its prognostic impact on OS (relative risk, 1.669; 95% CI, 1.031–2.702; *P* = 0.037; Table 3) and PFS (relative risk, 1.877; 95% CI, 1.227–2.872; *P* = 0.004; Table 3) in the set of overall patients. Among the other variables studied, age and LDH levels were shown to be independent prognostic factors for OS, while advanced Ann Arbor stage independently predicted inferior PFS.

## Discussion

Pathogenesis and survival are thought to be influenced by a deficiency of host immunity. Survival outcomes in lymphoma patients have been shown to be influenced by immune cells in the tumor microenvironment, including the tumor infiltrating lymphocytes, and tumor associated cells of the monocytic lineages [7], [8], [17], [18]. A recent gene expression profile study identified “stromal-2” gene signatures in DLBCL patients, which are reflective of the tumor microenvironment, and are associated with clinical outcomes in DLBCL [8]. A prognostic model has been recently proposed, incorporating two genes reflecting tumor and the microenvironment. The TNFRSF9 gene (tumor necrosis factor receptor superfamily member 9), related to immune microenvironment, demonstrated a powerful influence on survival outcomes of DLBCL [19]. A recent study by Challa-Malladi M et al. showed that the pathogenesis of DLBCL is related to the evasion of immune-recognition and the defective expression of cell-surface molecules, which facilitated the escape of DLBCL cells from immune-survillance [20].

LMR was recently shown to be an independent prognostic indicator in Hodgkin’s lymphoma (HL) [21], [22]. However, until now, there has been limited data regarding the role of this integrated biomarker LMR in the prognosis of DLBCL in the rituximab era. In this study, we evaluated the impact of baseline LMR, and integrating AMC and ALC (surrogate biomarkers of tumor microenvironment and host immunity), on the treatment response and prognosis in DLBCL patients with standard R-CHOP therapy. We used ROC curve analysis to generate an objective and reliable LMR cutoff value for survival analysis in the training set. Low LMR at diagnosis was associated with adverse clinical features, including poor performance status, elevated LDH level, advanced stages, and high IPI score.,LMR was found to be an effective independent prognostic factor for OS and PFS of DLBCL in the testing set as well as the set of overall patients. More than one half of the patients were categorized in the low-risk IPI group, and low LMR was also useful in identifying patients with low survival in this low-risk IPI category.

Baseline AMC was recently reported as an adverse prognostic factor in DLBCL, HL, and follicular lymphoma (FL) [14], [21], [22], [23]. Our results suggested that AMC at diagnosis was also correlated with survival in DLBCL patients treated with R-CHOP therapy. Genomic studies previously showed that myeloid-lineage cells in a tumor microenvironment predicted survival in DLBCL patients [8]. An elevated ratio of CD14+ monocytes without HLA expression was reported to be significantly related to aggressive clinical behaviors in DLBCL [24]. These peripheral blood monocytes suppressed host immunity by inhibiting the recall response and proliferative ability of interferon-γ, and impairing the differentiation ability of dendritic cells. T-cell lymphomas are characterized by heavy infiltration of myeloid-derived cells (MDCs), including monocytes and their progeny, within the tumor microenvironment. Monocytes have also been shown to promote the growth and proliferation of T or NK lymphoma cells [25], [26].

Lymphopenia has been acknowledged as a factor adversely influencing the international prognostic score (IPS) of HL and the predictive role of ALC has been established in DLBCL and other subtypes of NHL [12], [13], [27], [28], [29]. Our results showed a similar relationship between baseline ALC and survival in DLBCL patients. ALC at diagnosis was considered an indicator of host immunity, and lymphopenia prior to initial treatment might be a sign of preexisting immunodeficiency [30], [31]. Low CD4+ T lymphocyte counts were noted to be related to chemotherapy toxicity [32], suggesting that the reduction of ALC might involve all subsets of lymphocytes.

Although both AMC and ALC were associated with survival outcomes in DLBCL, they seemed to have a limited ability to identify high-risk patients. In this study, we showed that both AMC and ALC were related to survival outcomes in DLBCL. However, our multivariate analysis showed that only LMR was a prognostic factor for OS and PFS. LMR is easily derived from a simple CBC blood test, and it is both technically and financially feasible to conveniently apply this protocol in routine clinical practice. The major limitation of this research is that it is a retrospective study. We were therefore unable to control for underlying positive or negative biases during the treatment or selection of patients. Given the limitation of its retrospective nature, we believe that it is important to validate these data in future prospective studies.

In conclusion, LMR at diagnosis showed promise as a prognostic factor of survival outcomes in DLBCL patients receiving R-CHOP therapy. This biomarker, integrating AMC and ALC, can be used as a simple surrogate indicator of tumor microenvironment and host immunity. Further studies are required to more fully understand the relationship between systemic immune suppression and prognosis of DLBCL in the rituximab era.

## Methods

### Ethics Statement

Written informed consent was obtained from all patients prior to treatment. This study was approved by the Institutional Review Board (IRB) of Sun Yat-Sen University Cancer Center, and was performed in accordance with the principles expressed in the Declaration of Helsinki.

### Patients and Staging

This study is a retrospective analysis of 438 patients, admitted and treated during the period between April 2002 and December 2009. Written informed consent for use of the medical records stored in the hospital, was obtained from all patients. We acquired separate consent for use of these for medical research. All eligible cases were selected consecutively. Inclusion criteria were: (i) presence of histologically confirmed diagnosis of DLBCL with positive expression of CD20, according to the WHO classification of Tumors of Haematopoietic and Lymphoid Tissues [1]; (ii) no previous treatment; (iii) receiving standard immunochemotherapy as first-line treatment: CHOP (cyclophosphamide, doxorubicin, vincristine, and prednisone) chemotherapy in combination with rituximab; (iv) no previous neoplasm or second primary malignancy; (v) no severe coincident diseases; (vi) availability of clinical information and follow-up data. The histologic diagnosis of DLBCL was retrospectively reviewed and confirmed by pathologists who were unaware of clinical outcomes. Patients with human immunodeficiency virus infection and those with primary central nervous system lymphomas were excluded.

Of the 438 patients, 200 patients were randomly assigned to the training set using a computer program, while the remaining patients were assigned to the testing set. Clinical data available prior to treatment included patient demographics, Eastern Cooperative Oncology Group (ECOG) performance status, physical examinations, systemic B symptoms, complete blood count, biochemical profiles, serum lactate dehydrogenase (LDH) level, number of extranodal involved sites, bone marrow findings, computed tomography (CT) scans of the thorax, abdomen, and pelvic cavity, or whole body positron emission tomography/computed tomography (PET/CT) scans. Absolute monocyte count (AMC) and absolute lymphocyte count (ALC) in peripheral blood were derived from the standard automated complete blood counts (CBC) which were done at diagnosis. All patients were staged according to the Ann Arbor staging system. The International Prognostic Index (IPI: stage, ECOG performance status, serum LDH, stage, extranodal sites) was evaluated as previously described [33].

### Treatment Modalities and Response Criteria

All 438 patients received standard CHOP regimen (cyclophosphamide, doxorubicin, vincristine, and prednisone) plus rituximab (R-CHOP) as first-line therapy. R-CHOP regimen consists of rituximab (375 mg/m^2^) on day 1; cyclophosphamide (750 mg/m^2^), doxorubicin (50 mg/m^2^), and vincristine (1.4 mg/m^2^; maximal dose, 2 mg) on day 2; and prednisone (100 mg/d) on days 2 to 6. R-CHOP therapy was administered every 3 weeks as previously described [34]. Patients in this group received R-CHOP therapy for 3 to 8 cycles as first-line treatment. Residual disease, extranodal disease, or previously bulky disease were treated by radiotherapy followed by chemotherapy. Dose adjustment of chemotherapy, and the number of chemotherapy cycles were decided at the discretion of the physicians. Treatment response was evaluated based on the International Working Group Recommendations for Response Criteria for non-Hodgkin’s lymphoma [35], [36].

### Statistical Analysis

The selection of cutoff values of peripheral blood lymphocyte-to-monocyte ratio (LMR), AMC, and ALC for survival analysis was determined using receiver operating characteristics (ROC) curve analysis in the training set (n = 200). Survival outcomes were dichotomized into alive *versus* death in the ROC curve analysis. The correlation between LMR and clinical parameters was assessed by the chi-square test or Fisher’s exact test. The influence of LMR, AMC, and ALC at diagnosis on the survival outcomes of DLBCL was analyzed in the testing set (n = 238) and in the set of overall patients (n = 438). The Kaplan–Meier method was used to determine overall survival (OS) and progression-free survival (PFS). OS was defined as the duration from diagnosis until the date of death from any cause, or date of the last follow-up. PFS was measured as the duration from diagnosis until the date of first lymphoma progression, death from any cause, or date of the last follow-up. Survival curves were generated by the Kaplan–Meier method. The prognostic impact of different variables on survival was determined by multivariate Cox proportional hazards model. The two-tailed log-rank test was employed to estimate statistical difference. All P values of less than 0.05 were regarded as statistically significant. Statistical analysis was carried out using SPSS 16.0 software.
